# High-resolution PET imaging reveals subtle impairment of the serotonin transporter in an early non-depressed Parkinson’s disease cohort

**DOI:** 10.1007/s00259-020-04683-4

**Published:** 2020-02-04

**Authors:** Patrik Fazio, Daniel Ferreira, Per Svenningsson, Christer Halldin, Lars Farde, Eric Westman, Andrea Varrone

**Affiliations:** 1 Center for Psychiatry Research, Department of Clinical Neuroscience, Karolinska Institutet, & Stockholm Health Care Services, RegionStockholm, Karolinska University Hospital, SE-17176, R5:02, Visionsgatan 70A, Stockholm, Sweden; 2 Department of Neurology, Karolinska University Hospital, Stockholm, Sweden; 3 Department of Neurobiology, Care Sciences and Society, Center for Alzheimer Research, Division of Clinical Geriatrics, Karolinska Institutet, Stockholm, Sweden; 4 Section of Neurology, Department of Clinical Neuroscience, Karolinska Institutet, Stockholm, Sweden; 5 Department of Neuroimaging, Centre for Neuroimaging Sciences, Institute of Psychiatry, Psychology and Neuroscience, King’s College London, London, SE5 8AF UK

**Keywords:** Parkinson’s disease, The serotoninergic system, Raphe nuclei, Functional connectivity/graph analysis

## Abstract

**Purpose:**

The serotonin transporter (SERT) is a biochemical marker for monoaminergic signaling in brain and has been suggested to be involved inthe pathophysiology of Parkinson’s disease (PD). The aim of this PET study was to examine SERT availability in relevant brain regions in early stages ofnon-depressed PD patients.

**Methods:**

In a cross-sectional study, 18 PD patients (13 M/5F, 64 ± 7 years, range 46–74 years, disease duration 2.9 ± 2.6 years; UPDRS motor 21.9 ± 5.2) and 20 age- and gender-matched healthy control (HC) subjects (15 M/5F, 61 ± 7 years, range 50–72 years) were included. In a subsequent longitudinal phase, ten of the PD patients (7 M/3F, UPDRS motor 20.6 ± 6.9) underwent a second PET measurement after 18–24 months. After a 3-T MRI acquisition, baseline PET measurements were performed with [^11^C]MADAM using a high-resolution research tomograph. The non-displaceablebinding potential (BP_ND_) was chosen as the outcome measure and was estimated at voxel level on wavelet-aided parametric images, by using the Logan graphical analysis and the cerebellum as reference region. A molecular template was generated to visualize and define different subdivisions of the raphe nuclei in the brainstem. Subortical and cortical regions of interest were segmented using FreeSurfer. Univariate analyses and multivariate network analyses were performed on the PET data.

**Results:**

The univariate region-based analysis showed no differences in SERT levels when the PD patients were compared with the HC neither at baseline or after 2 years of follow-up. The multivariate network analysis also showed no differences at baseline. However, prominent changes in integration and segregation measures were observed at follow-up, indicating a disconnection of the cortical and subcortical regions from the three nuclei of the raphe.

**Conclusion:**

We conclude that the serotoninergic system in PD patients seems to become involved with a network dysregulation as the disease progresses, suggesting a disturbed serotonergic signaling from raphe nuclei to target subcortical and cortical regions.

**Electronic supplementatary material:**

The online version of this article 10.1007/s00259-020-04683-4

## Introduction

Parkinson’s disease (PD) is a multidomain neurodegenerative disease with a wide phenotypic expression. Non-motor symptoms such as depression, cognitive decline, sleep disorders, and dysautonomia [1] have a relevant impact on the quality of life and prognosis of the patients. The clinical expression of such symptoms is likely related to the accumulation of misfolded proteins (alpha-synuclein, tau, and amyloid) [2, 3] as well as to patterns of cortical atrophy [4]. However, molecular changes in different neurotrasmitter systems are also related to the expression of clinical phenotypes.

The implication of the serotoninergic system has been hypothesized based on evidences from different postmortem studies. An early pathological involvement of the brainstem has been described by the Braak staging system [5]. More specifically, the involvement of different serotoninergic nuclei of the raphe (dorsal, medial, and caudal) has been reported in postmortem brainstem samples from PD patients [6]. Another postmortem study demonstrated that different serotonin markers including its transporter (SERT) were reduced in PD patients as compared with healthy controls, with a prevalent involvement of the caudate over the putamen [7].

In vivo studies have contributed to better understand the involvement of the serotonin system in PD. A PET study in the 1-methyl-4-phenyl-1,2,3,6-tetrahydropyridine (MPTP) non-human primate model has shown that the injury of the serotoninergic nerve terminals with 3,4-methylenedioxy methamphetamine (MDMA) altered rigidity and abolished l-dopa-induced dyskinesia and neuropsychiatric like behaviors [8]. Molecular imaging studies in PD patients have later examined the availability of serotoninergic targets in relation to motor and non-motor symptoms and behavior. In the early phases of PD, dysregulation of serotoninergic innervation has been shown mainly in the caudate, thalamus, hypothalamus, and anterior cingulate cortex [9]. At later stages reductions were described in the putamen and within the insula, posterior cingulate cortex, and prefrontal cortex [9]. Only advanced PD patients showed significant reductions in the ventral striatum, raphe nuclei, and amygdala [10]. Specific and distinct serotonergic dysfunctions have been detected with PET in PD in association with depression [11], apathy and anxiety [12, 13], and sleep disturbances [14]. Moreover the serotonin system is implicated in the development of levodopa-induced dyskinesia [15]. Findings related to SERT availability in the brainstem are so far not conclusive and a clear impairment in early phases has not been consistently reported [9, 11, 13, 16, 17]. More recently, alterations of the serotonergic transmission in the brainstem have been described in premotor A53T alpha-synuclein mutation carriers (A53T SNCA) [18]. In our view, the capability of evaluating serotoninergic targets in the different raphe nuclei has so far been challenged by the limited resolution of the PET system for such small structures and by the fact that those nuclei cannot be easily defined or segmented on standard MRI data.

With this cross-sectional and longitudinal study, we aimed at investigating the role of SERT in early non-depressed PD patients. We were specifically interested to examine the impairment of SERT availability in the brainstem nuclei and in relevant projection areas using a high-resolution PET system. In order to accomplish our objective, a methodological approach that we have recently developed and validated for the quantification of SERT availability in the brainstem [19] was used. In addition to the traditional region-based appoach, we also aimed to characterize network characteristic with explorative graph analysis. For both approaches, we compared early non-depressed PD patients with age- and sex-matched healthy controls cross-sectionally, and we followed PD patients longitudinally after 2 years.

## Methods

### Participants

We aimed at including 20 PD patients and 20 healthy controls between 45 and 80 years of age. These cohorts have been also examined with dopamine transporter PET imaging and the data have been reported separately [20]. All subjects were healthy according to medical and psychiatric history, physical examination, laboratory assessment, and radiological examination of magnetic resonance imaging (MRI). We targeted healthy PD patients with a clinical diagnosis of PD according to the UK Brain Bank criteria, with Hoehn and Yahr stages 1 to 2, drug-naïve (de novo) or on treatment with l-dopa, catechol-O-methyltransferase (COMT) inhibitors, monoamine oxidase B (MAO-B) inhibitors, and/or dopamine receptor agonists. PD patients were primarily recruited from the Department of Neurology at Karolinska University Hospital Huddinge (Sweden), by advertisement in a local newspaper and at local Parkinson’s patient association. Healthy subjects were recruited by advertisement in a local newspaper. All subjects were assessed at inclusion at the Karolinska PET center. No medications with significant action on the dopamine, serotonin, or noradrenergic transporter were allowed for healthy subjects participating in this study. Previous and current use of antidepressant and antipsychotic medications was an exclusion criterion. PD participants abstained from treatment with l-dopa, COMT inhibitors, MAO-B inhibitors, and/or dopamine receptor agonists at least 12 h prior to PET examination. Urine drug and breath alcohol tests were conducted before each examination. Participants not able to abstain from smoking or nicotine use overnight were also excluded. All PD subjects were offered to come back after 18–24 months for a follow-up PET measurement. The study was approved by the Ethics Committee of the Stockholm Region and by the Radiation Safety Committee of the Karolinska University Hospital, Solna, Stockholm, Sweden. After a detailed explanation of the procedures and visits, the subjects signed written consent to participate in the study.

### Preparation of [^11^C]MADAM

[^11^C]MADAM was synthesized by methylation of 2-((2-((dimethylamino)methyl)phenyl)thio)-5-iodophenylamine (ADAM) and radiolabeled using ^11^C-methyl triflate, as previously reported [21].

### PET imaging procedures and analysis

PET measurements were conducted using a high-resolution research tomograph (HRRT) (Siemens Molecular Imaging) after a bolus injection of [^11^C]MADAM (injected radioactivity 390.9 ± 36.3 MBq). Details for specific activity and injected mass are reported in supplementary Table 1. To prevent for head motion during the PET measurement, an individual plaster helmet was made for each subject [22]. A 6-min transmission scan using a rotating ^137^Cs source was first acquired for attenuation correction. Emission data were acquired in list mode for a period of 93 min. Dynamic images were reconstructed in a series of 31 frames (4 × 15 s, 4× 30 s, 6 × 60 s, 6 × 180 s, 11 × 360 s) using three-dimensional ordinary Poisson ordered subset expectation maximization (OP-3D-OSEM), including modeling of the system’s point spread function (PSF). This procedure corresponds to a resolution of approximately 2 mm [23]. Images were also corrected for motion with a post-reconstruction frame-to-frame correction realignment algorithm [24]. Parametric images of non-displaceable binding potential (BP_ND_) were generated using the wavelet-aided parametric imaging (WAPI) algorithm. The WAPI algorithm utilizes a wavelet-based denoising approach in order to reduce the high voxel-to-voxel noise level present in reconstructed dynamic PET data. This approach has been shown to successfully reduce the high voxel- to-voxel noise level typically present in raw dynamic PET data. [25]. WAPI utilizes the Logan graphical analysis approach in order to calculate BP_ND_ in each voxel. The cerebellum was used as the reference region because it is a suitable reference tissue for [^11^C]MADAM [26].

### MRI acquisition and processing

T1-weighted 3-dimensional images were acquired for co-registration with PET images and to obtain normalization parameters to the standard MNI space. A T2-weighted sequence was included to rule out pathology. All images were co-registered to PET space and segmented into gray matter (GM), white matter (WM), and cerebrospinal fluid (CSF) compartments using SPM5 (Welcome Department of Cognitive Neurology, University College London). MRI data were acquired with a GE MR750 3-Tesla system (IR-SPGR sequence; voxel resolution of 1 × 1 × 1 mm, TI = 450 ms, TE = 3.18 ms, TR = 8.16 ms). The MRI acquisitions were performed on average 2 weeks prior to the PET examination. The T1-weighted images were processed with the FreeSurfer 5.1 image analysis suite in order to obtain a subset of regions of interest. Technical details of the FreeSurfer processing are described in prior publications [27]. Briefly, this processing includes motion correction and averaging of multiple volumetric T1-weighted images [28], removal of non-brain tissue using a hybrid watershed/surface deformation procedure [29], whole brain segmentation [30], segmentation of the subcortical structures [31], and intensity normalization [32]. Quality checks were performed on FreeSurfer output of each individual.

### Definition of regions of interest

Left and right caudate, putamen, globus pallidus, and thalamus as well as anterior and posterior cingulate regions of interest (ROIs) were obtained from the segmentation and parcellation routines of FreeSurfer performed on T1-weighted images. The cerebellar gray matter was manually delineated. The brainstem ROIs for the three raphe nuclei (medial, anterior, and posterior raphe) were obtained by using a molecular-based PET template generated with 3-T MR images and parametric images of [^11^C] MADAM from 10 healthy control subjects of the same cohort. An automated MR-based two-step normalization procedure using various tools of the FMRIB Software Library (FSL) was employed to obtain accurate brainstem normalization and to obtain individual co-registration and normalization parameters as previously described [19]. All ROIs were co-registered to PET space and then projected onto the *BP*_ND_ maps (parametric images) in order to obtain average ROIs regional *BP*_ND_ values with the inverse normalization and co-registrion matrix [20].

### Network analysis

**Fig. 1:**
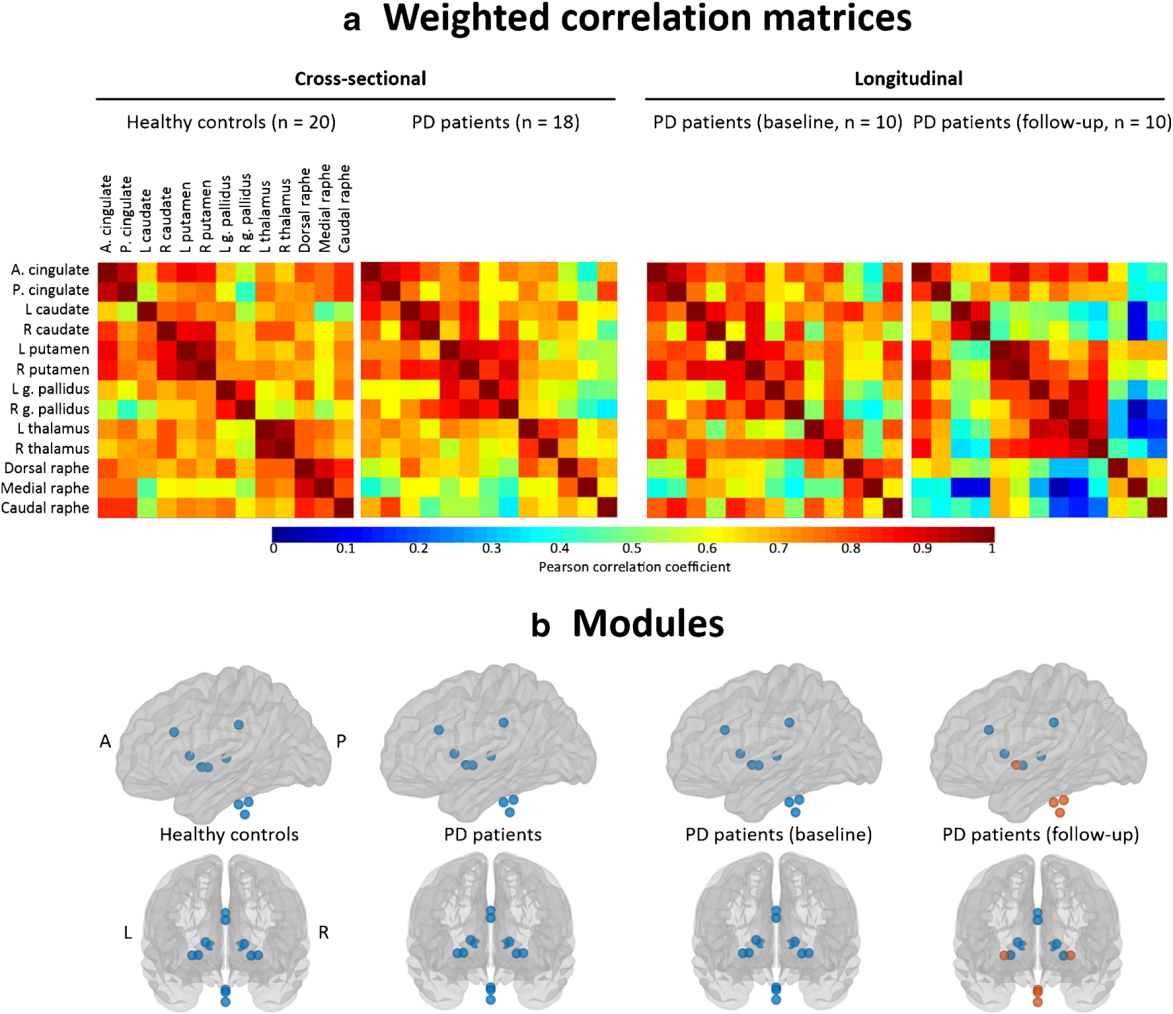
**a**, **b** The upper panel (**a**) shows weighted correlation matrices based on SERT binding potential values (*BP*_ND_) of PD patients and HC at baseline, as well as for the 10 PD patients with longitudinal data (the same 10 PD patients both at baseline and follow-up assessments for longitudinal analysis). The lower panel (**b**) shows brain modules at baseline and at follow-up. Brain modules I in blue, module II in orange. A anterior, P posterior, L left, R right, PD Parkinson’s disease, g. pallidus globus pallidus

Brain networks can be built by using a set of nodes connected by edges. We performed this analysis using BRAPH (http://braph.org) [33]. Regarding the nodes, we used all the ROIs described above, giving a total of 13 functionally relevant regions for SERT distribution in relation to projecting and projection areas of the serotonin system. Regional SERT BP_ND_ values from these regions were extracted. Regarding the edges, Pearson correlations between all pairs of anatomical regions were used and recorded in a 13 × 13 matrix for each group (Fig. 1a). These matrices were symmetric, which corresponds to undirected graphs. We applied a range of threshold densities (i.e., fraction between significant correlations and all possible correlations) to the group matrices to generate binary networks so that correlations above the threshold are set to 1 and those below the threshold are set to 0. We used a set of threshold densities ranging from Smin = 20% to Smax = 35%, sampled in steps of 1% to threshold each group’s respective correlation matrix, ensuring exclusion of disconnected networks (Smin < 20%) and random network topology (Smin > 35%). In the present study, we were interested in investigating global integration and segregation properties of the serotoninergic network, as well as its modular organization. Integration measures reflect the capacity of the brain to rapidly combine information from distributed brain regions. Segregation measures reflect the biologically meaningful feature of the brain to enable highly specialized processing through densely interconnected communities of regions. To that end, we analyzed the average global efficiency, the average local efficiency, the transitivity, and the modularity. Global efficiency is a measure of network integration, providing information about the network’s ability to rapidly incorporate information from distinct anatomical regions. Local efficiency is similar to the average global efficiency but is restricted to a given node and its immediate neighbors. Transitivity and modularity are segregation measures. The transitivity is normalized by the whole network [34], and it measures the extent to which the nodes surrounding a certain node are also connected to each other [35]. The modularity reflects the presence of modules or communities of regions in a network. Networks with high modularity have a well-defined modular structure with a high number of within-module connections and a low number of between-module connections [36]. Additionally, we performed modular analyses by using the Newman algorithm [37], based on weighted networks (i.e., the correlation matrices before binarization) with a gamma value of 1 [38]. While the modularity is a sophisticated quantitative measure that reflects the existence of communities of regions within a network [39], it cannot provide any information about the specific belonging of brain regions to the actual communities. This can in turn be qualitatively assessed by modular analyses as shown in Fig. 1b. The formulae used to calculate all these graph measures are provided by Rubinov and Sporns [40].

### Statistics

According to our study aims, we performed statistical analyses at two levels: univariate analyses across 7 ROIs and multivariate network analyses across 13 ROIs. The number of ROIs for the univariate analyses was reduced by combining right and left estimations in order to minimize the number of statistical comparisons. A series of independent samples *t* tests with BP_ND_ values of [^11^C]MADAM were conducted, comparing PD and healthy control groups for each outcome measure. The Bonferroni correction was used for multiple testing with an alpha threshold of 5% (two-tailed). For longitudinal analyses, we estimated the percent change from baseline [^11^C]MADAM BP_ND_ in all 7 ROIs. A series of dependent samples *t* tests were also used to measure differences in the SERT availabilities from baseline to the follow-up assessment. Regarding the multivariate network analyses, between-group comparisons of graph measures were conducted through 1000 nonparametric permutations at a range of network densities (min = 20% to max = 35%, in steps of 1%). The 95% confidence intervals of each distribution were used as critical values for testing of the null hypothesis at an alpha threshold of 5% (two-tailed). All network analyses were conducted using BRAPH (http://braph.org, [33]).

## Results

### Participants

A total of 52 subjects were screened (24 PD patients and 28 healthy controls). Four PD patients and 8 healthy control (HC) subjects were excluded for not fulfilling the selection criteria. One PD patient was excluded because of low-injected activity of [^11^C]MADAM, and another PD patient was excluded from the cross-sectional and longitudinal part of the study because of low quality of segmentation. Demographic and clinical data for Parkinson’s disease (PD) patients, healthy controls, and PD patients at follow-up are shown in Table 1.

**Table 1:** Healthy controls and Parkinson’s patient characteristics at baseline and at follow-up

	Healthy controls	Early PD patients	PD patients after 2 years
*n*	20	18	10
Sex	5F/13M	5F/13M	3F/7M
Age	61 ± 7 (50–72)	64 ± 7 (46–74)	66 ± 7 (48–74)
MMSE	29 ± 0.6 (28–30)	29 ± 1 (27–30)	NA
Disease duration (year)	NA	2.9 ± 2.6 (0.3–12)	4.9 ± 3 (2–14)
UPDRS motor	NA	22 ± 5 (11–31)	21 ± 7 (12–34)
Hoehn and Yahr	NA	1.5 ± 0.5 (1–2)	1.7 ± 0.5 (1–2.5)
LED count	NA	370 ± 255 (0–940)	656 ± 500 (120–1600)

*UPDRS motor* unified Parkinson’s disease rating scale part III, *MMSE* mini-mental state, *LED count* levodopa equivalent

### Region-based analysis: cross-sectional

**Fig. 2:**
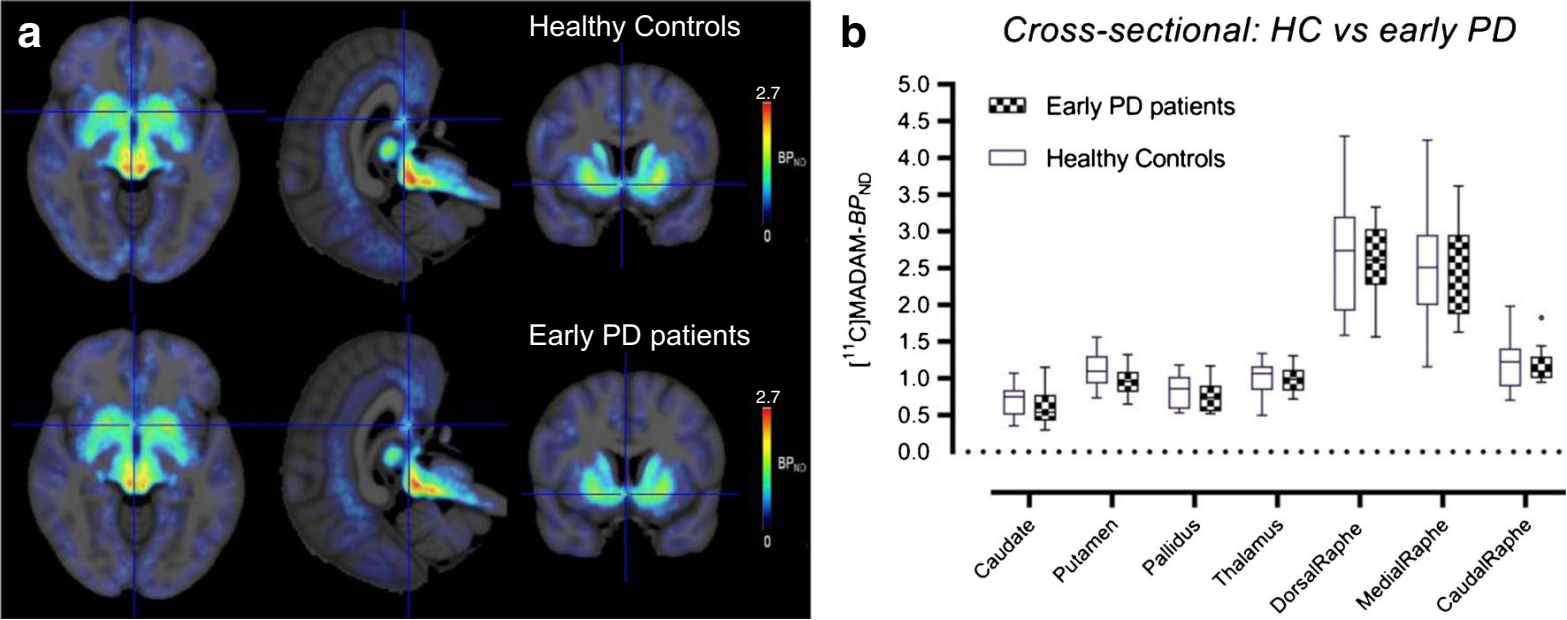
**a** Mean parametric images of BP_ND_ from 20 healthy controls and 18 patients with Parkinson’s disease overlaid on MR images. **b** Boxplots with Tukey whiskers representing regional binding potentials (BP_ND_) values obtained with [^11^C]MADAM in the same cohorts

In our regional analysis, we observed that the distribution of SERT availabilities was representative of the known distribution of SERT in the brain with higher values in the dorsal and median raphe nuclei followed by the caudal raphe and other subcortical regions. In early PD patients, SERT availabilities in subcortical regions were lower than HC by 11.2% in the caudate, 11.8% in the putamen and 7.7% in the pallidus (Fig. 2). However, none of these differences was statistically different from that of the controls. A trend was obtained for the putamen (*p* = 0.06). In all subdivisions of raphe nuclei (dorsal, median, rostral), SERT availabilities were lower in PD than in HC only by 2.8% on average (not significant) (Fig. 2).

### Region-based analysis: longitudinal

**Fig. 3:**
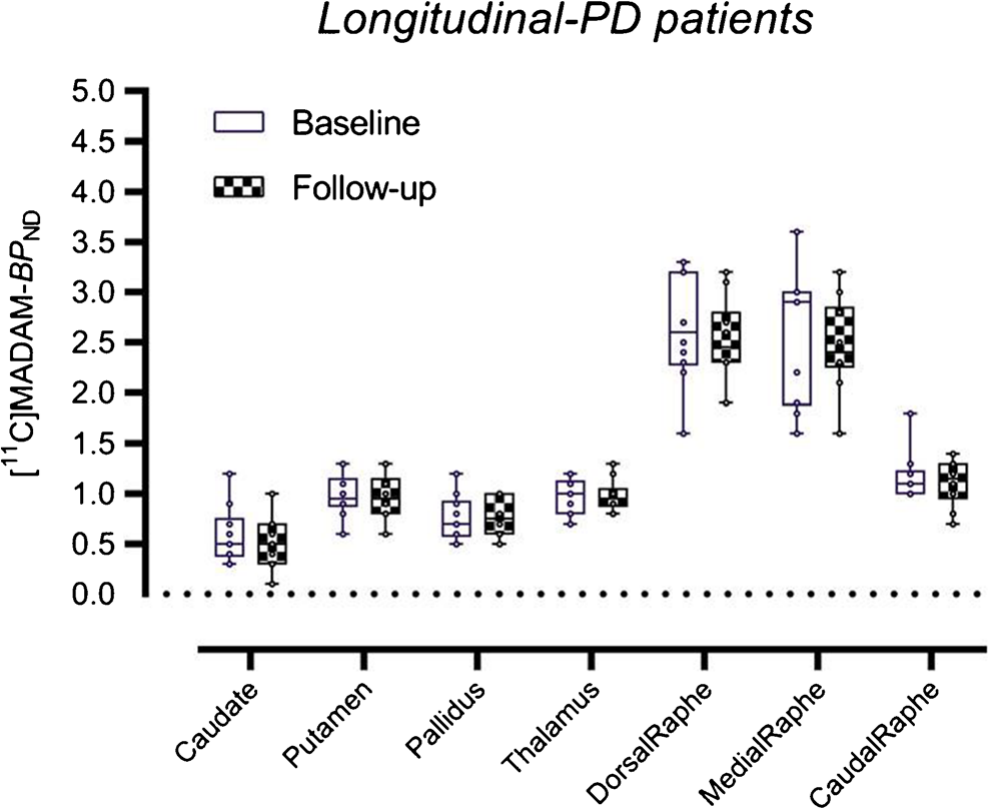
Boxplots with Tukey whiskers representing regional binding potential values (BP_ND_) obtained with [^11^C]MADAM PET in10 PD patients at baseline and at follow-up after 2 years

Only the PD patients received a follow-up PET scan; hence, longitudinal analyses were only conducted in the PD patients (*n* = 10). We observed that, at follow-up, all patients showed higher medication intake reflected by an evident increase of LED values and almost unaltered motor scores as assessed by the UPDRS. After 2 years of follow-up, we found a 9.8% decrease in the caudate, 3.5% in the putamen, 3% in the dorsal raphe, 3.7% in the medial raphe, and 5.5% in the caudal raphe as compared with baseline data of the same 10 PD patients (Fig. 3). In the thalamus and in the globus pallidus, BP_ND_ values at baseline and after 2 years were almost identical. The observed differences were not statistically significant before and after Bonferroni’s correction.

### Network analysis: cross-sectional

PD patients did not differ from HC in network measures at baseline. Inspection of the correlation matrices shows rather homogeneous associations across all regions in the HC, whereas in PD patients, the three nuclei of the raphe were weakly connected to the cingulate and subcortical areas (Fig. 1a). Regarding modular analyses, only one module including all 13 ROIs was found both in HC and PD patients (Fig. 1b).

### Network analysis: longitudinal

**Fig. 4:**
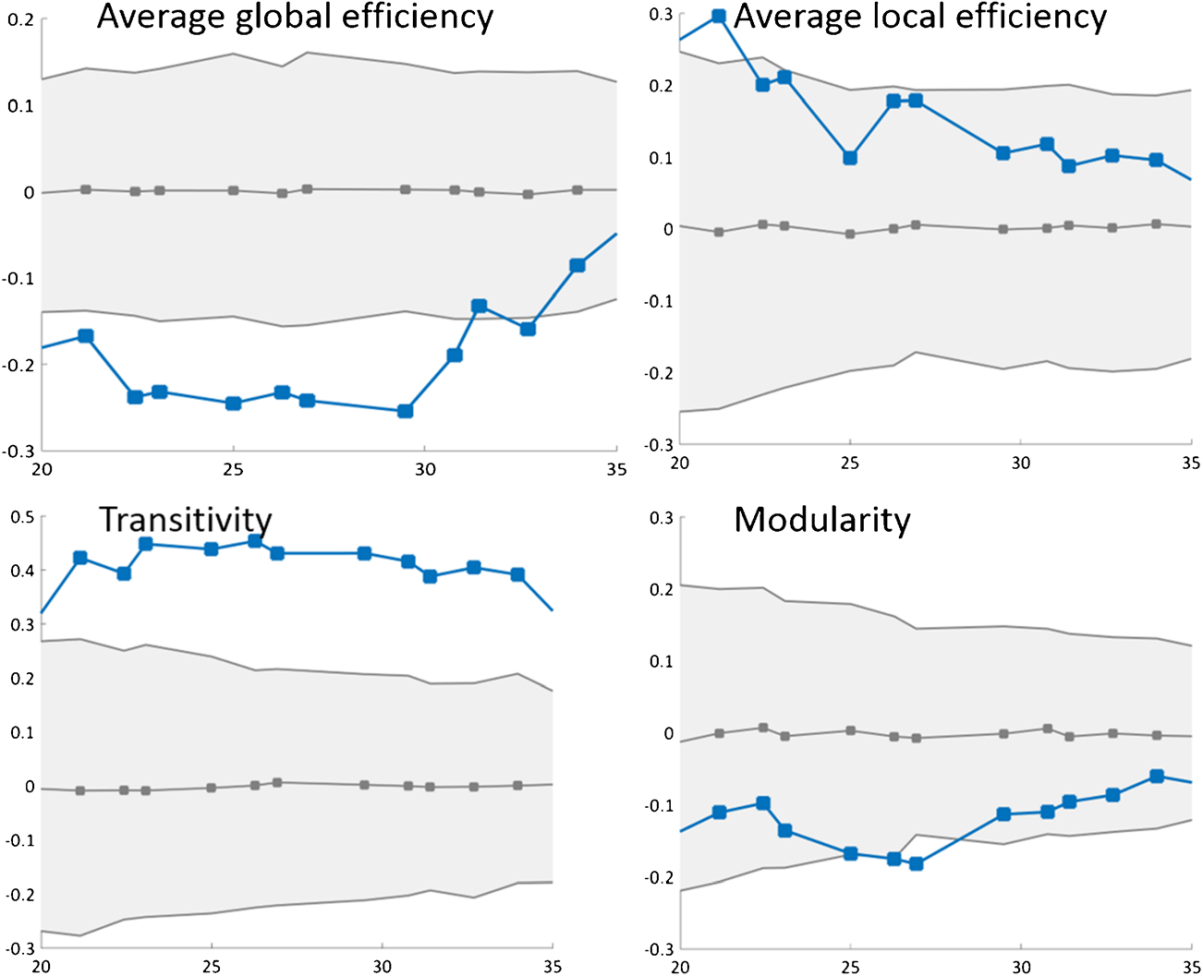
Comparison of network measures (baseline vs follow-up in Parkinson’s disease patients). Network densities are displayed on the *x*-axis from min = 20% to max = 35%, in steps of 1%. Between-group differences in the global graph measures are displayed on the *y*-axis. The plots show the lower and upper bounds (gray solid lines) of the 95% confidence intervals (CI) (gray shaded areas) as a function of density. The blue dotted lines show the differences between baseline and follow-up and when falling outside the CI they indicate that the difference was statistically significant at *p* < 0.05. The gray dotted lines in the middle with values around zero indicate the mean values of the difference in network measures between the randomized groups after permutation tests

In contrast to the cross-sectional analyses, we found significant differences between PD patients and HC in network measures (Fig. 4). After 2 years of follow-up, PD patients showed a prominent reduction in the average global efficiency and a prominent increase in transitivity. Inspection of the correlation matrices shows a reduction in the associations of the 3 raphe nuclei with the cingulate regions and all the subcortical regions (except for the putamen) after 2 years of follow-up (Fig. 1a). The modular analysis further highlighted this finding by showing that after 2 years of follow-up, the modular organization in PD patients changed from one single module including all 13 ROIs to two modules (Fig. 1b). The first module includes the three nuclei of the raphe and the putamen, whereas the second module includes the cingulate regions and all other subcortical regions. No consistent differences across densities were observed for the average local efficiency and the modularity measures (Fig. 4).

## Discussion

In the present study, we investigated the serotoninergic system of early non-depressed PD patients using the radioligand [^11^C]MADAM and high-resolution PET imaging. We employed a methodology specifically developed to quantity the SERT in the caudal, rostral, and medial raphe nuclei based on a standard VOI template. In addition, we quantified SERT availability in the major projection regions of the cortex and striatum. We found that SERT availability was relatively preserved in the early non-depressed PD patients at baseline when compared with age- and gender-matched HC. With disease progression after 2 years of follow-up, we found prominent changes in integration and segregation properties of the serotoninergic network, leading to changes in the modular organization of the network.

To our knowledge, this is the first study in PD patients that uses a semi-automatic procedure to quantify SERT availability in different subdivisions of the raphe nuclei. Other methods based on manual definition of the region on MRI images are subjective and might lead to an erroneous definition and localization of the structures [41]. Our method is user-independent, less affected by noise, since it is based on parametric images and not on regional time activity curves analysis, and less dependent on partial volume effects. Altogether, we believe that our method provides a robust quantification of the SERT in the brainstem.

The possibility to quantify the different portions of the raphe nuclei (dorsal, medial, and caudal) is relevant for the study of the neurobiology of the serotonin system in PD [42]. Indeed, the raphe nuclei are the main sources of serotonin projection from the brainstem to different brain regions, including the basal ganglia, thalamus, hippocampus, amygdala, and cortex, as well as to the spinal cord. A detailed description of dysregulation of the serotonin system at different levels (brainstem/subcortical/cortical) might shed some light on molecular and pathophysiological aspects related to the ongoing degeneration process.

Our cross-sectional comparisons suggest a relative preservation of SERT in early non-depressed PD patients as compared with HC, which is in agreement with previous findings [9, 16, 17]. On the other hand, these findings are not in agreement with a recent article that have described clear and extensive dysregulations of serotonergic signaling in the brainstem of different presymptomatic and symptomatic PD cohorts supporting a “Braak-like” spreading of serotoninergic alterations [18]. This disagreement might be explained by methodological differences such as the use of PET systems with different spatial resolutions (HRRT vs PET-CT), different radioligands ([^11^C]MADAM vs [^11^C]DASB), and different definitions of brainstem subnuclei (semi-automatic vs manual). However, we cannot exclude that the two studies included different PD cohorts with different degrees of SERT impairment resulting in different ranges of binding potential values. Future studies in other PD cohorts are thus warranted in order to clarify these contradictory results.

In the longitudinal evaluation, we observed a relative longitudinal preservation of SERT binding in the region-based analysis (univariate). However, we found a prominent disruption of the serotoninergic network in the multivariate analysis (graph theory). We observed signs of disconnection between brainstem, subcortical regions, and the cingulate as PD progresses. The reduced average global efficiency could contribute to an abnormal segregation of the whole network (increased transitivity), leading to loss of the single module organization (emergence of two modules after 2 years of follow-up). The modular analysis showed that the three nuclei of the raphe and the putamen clustered together demarcating themselves from all other regions in the network after 2 years of follow-up.

The different results obtained by the univariate ROI analysis and the multivariate network analyses highlight that subtle changes can be better captured when investigating patterns in the data. Subtle changes can be diluted in univariate analysis at the group level due to variability across individuals. This has been discussed earlier in terms of the superiority of network analyses to detect very early brain changes in network topology of PD patients despite less or no overt changes in cortical thickness or volume using mass univariate analyses [43]. The same finding has been reported in other disorders such as Alzheimer’s disease [38, 44].

The results obtained with the univariate analysis in the cross-sectional part of the study indicate that the brainstem nuclei, enriched in SERT protein, are relatively preserved. In that perspective, our PD cohort does not seem to support the ascending progression of the disease as described by Braak [45]. The same cohort has also been examined with [^18^F]FE-PE2I, a dopamine transporter protein (DAT) radioligand [20]. In the cross-sectional part of that study, we observed significant molecular dopaminergic alterations in the midbrain (the substantia nigra) at the same disease time point. Taken together, these results confirm the “known” caveats of the Braak staging system since, in many cases, it does not match the progression of the clinical symptoms of PD [46, 47]. In terms of pathophysiology and implications, the results obtained in the longitudinal section indicates that at early stages in PD, the serotonergic system seems to be affected showing a disconnection of Raphe nuclei from projecting regions in the graph analyses. Axonal degenerations and loss of synaptic proteins and efficiency might account for the “disconnection” detected in this study at all 3 levels (brainstem/subcortical/cortical regions) [48, 49].

This study has some limitations. We do not have a complete clinical evaluation of PD patients at follow-up since the study was mainly designed as a follow-up PET study. We observed a large variation in terms of SERT availability values in the brainstem subnuclei, both in PD patients and in control subjects. This could indicate the presence of factors not controlled by the design of the study that could have influenced the binding patterns to SERT (e.g., sleep disorders [14], apathy or anxiety [13], and the presence of the brain-derived neurotropic factor (BDNF) val66met polymorphism [50]). Moreover, despite that SERT availabilities which did not correlate with the amount of daily dopaminergic treatment (levodopa equivalent), we cannot exclude that the chronic exposure to levodopa may influence the serotoninergic neurotransmission and the binding of [^11^C]MADAM to the SERT protein [51, 52]. On the other hand, we can notice that our PD patients were substantially stable in their UPDRS motor scores after 2 years. The relative stabilization of the UPDRS motor scores may be related to the increased amount of dopamine replacement therapies that was almost doubled but can also imply a non-malignant phenotype of the disease with optimal response to dopaminergic therapies. Finally, because of the sample size, the selection of ROIs in the univariate analysis as well as in the network analysis was restricted to a number close to the number of subjects. Therefore, the network analysis is exploratory and the current findings should be confirmed in future studies.

## Conclusion

The findings of this study indicate that the serotoninergic system might become involved in PD patients as the disease progresses. This finding was only captured on network measures, but not on direct regional binding, suggesting connectivity changes before overt depletion can be detected in the serotonergic system. Confirming our current findings in other PD cohorts is warranted in order to encourage the use of antidepressants at early stages of PD.

## Electronic supplementary materials

ESM 1 (DOCX 13.8 kb)
