# Supplementary material for: High-resolution PET imaging reveals subtle impairment of the serotonin transporter in an early non-depressed Parkinson’s disease cohort
Source: Eur J Nucl Med Mol Imaging. 2020 Feb 4;47(10):2407–16. doi: 10.1007/s00259-020-04683-4 (PMC7396398; doi:10.1007/s00259-020-04683-4)
Supplement: Supplementary file 1 — (DOCX 13.8 kb) [file 259_2020_4683_MOESM1_ESM.docx]

|  |  |  |  |  |  |
| --- | --- | --- | --- | --- | --- |
|  |  | *Healthy controls* | *Early PD patients* | *PD patients after 2 years* |  |
|  | *n* | 20 | 18 | 10 |  |
|  | *Inj dose* | 374±38 (262-410) | 373±46 (259-417) | 392±24 (345-419) |  |
|  | *(MBq)* |  |  |  |  |
|  | *Specific Activity* | 260±216(84-1100) | 241±204(32-946) | 217±64(106-302) |  |
|  | *(GBq/µmol)* |  |  |  |  |
|  | *Mass Injected* | 0.4±0.3(0.4-1.32) | 0.6±0.7(0.3-2.77) | 0.5±0.2(0.35-1) |  |
|  | *(µg)* |  |  |  |  |
|  |  |  |  |  |  |

Supplementary table 1. Details of [^11^C] MADAM injections (Injected dose, Specific Activity and Mass injected) in healthy controls and Parkinson’s patients at baseline and at follow-up
